# “Ten-year mixed method evaluation of prelicensure health professional student self-reported learning in an interfaculty pain curriculum”: a view on pain education

**DOI:** 10.1097/PR9.0000000000001031

**Published:** 2022-09-14

**Authors:** Anne-Priscille Trouvin

**Affiliations:** a Pain Medicine Department, Cochin University Hospital, Paris, France; b Medical School, Paris Cité University, Paris, France

**Commentary on:** Dale CM, Cioffi I, Murphy L, Langlois S, Musa R, Stevens B. Ten-year mixed-method evaluation of prelicensure health professional student self-reported learning in an interfaculty pain curriculum. PAIN Rep 2022;7:e1030.

I was intrigued and inspired by the paper entitled “Ten-Year Mixed Method Evaluation of Prelicensure Health Professional Student Self-Reported Learning in an Interfaculty Pain Curriculum.”^4^ The authors summarize the impact of their pain education curriculum in Canada on prelicensure health students over the course of 10 years.

This is the second paper from the same authors focused on their pain education curriculum. The first paper^3^ evaluated the evolution of students' pain knowledge and beliefs and approach to interprofessional collaboration in pain care before and after a 20-hour curriculum. This second paper measures the students' self-rating of knowledge acquisition and effective presentation methods and considers their feedback on the same curriculum.^4^

More than 10,000 students participated in the pain education curriculum over 10 years. In the first paper, Cioffi et al.^3^ measured the impact of the curriculum on students' pain knowledge and beliefs using a questionnaire developed by the local educational committee. However, the paper did not provide a copy of this questionnaire with publication, making it difficult to identify the complexity and relevance of the questions. Moreover, approximately half of the students enrolled in the curriculum each year completed the tests, so the impact on the other half remains unknown. The paper reported results that demonstrated a substantial decrease in the difference between pretest and posttest results over the 10 years, with a gain of only 1.8% in 2019. This finding raises the question of the relevance of the curriculum for students over the most recent years.

In the second paper, Dale et al.^4^ reported the students' feedback after completing the curriculum. Based on the same large cohort of more than 10,000 students over 10 years, the paper describes the feedback as good regarding self-rating of knowledge acquisition and satisfaction. It is important to note that this second paper only relies on self-declared results, and therefore, these reported outcomes are at risk of a social desirability bias.^3,4^

Given the burden of chronic pain worldwide, pain education is important in health care today and raising awareness about available pain curricula is a major publication end point. With an estimated prevalence of moderate to severe chronic pain of 19% in Europe,^1,13^ pain education is crucial. As identified by the American Institute of Medicine, one major hindrance to good quality of care for pain patients is “limited access to clinicians who are knowledgeable about acute and chronic pain—owing in part to the prevalence of outmoded or unscientific knowledge and attitudes about pain.”^11^ For 2 years until 2012, the International Association for the Study of Pain developed pain curricula outlines for health professionals as well as an interprofessional curriculum. In 2017, all 9 proposed curricula were revised and updated, and their content is accessible at the IASP web site.^8^ In Europe, the European Pain Federation (EFIC) has developed curricula on pain medicine, pain physiotherapy, pain nursing, and pain psychology.^5^

There are several considerations when developing pain education curricula, including time devoted to pain education and teaching and assessment methods. However, perhaps most important is defining the objectives of the curricula.

The first consideration is how much time to devote to pain education in university curricula. Despite international efforts to structure pain education and curricula, a median of 12 hours in Europe and 9 hours in the United States was allocated for pain medicine content in medical schools.^14^ With so few hours, topics addressed by pain curricula in different countries are variable and often not exhaustive of the IASP-recommended and EFIC-recommended topics. Health students note this lack of thorough pain education. For example, in one study reporting a cohort of Finnish students, the authors highlight that most of them considered their multidisciplinary pain education to be insufficient.^12^ As concluded by Shipton et al.^14^ in 2018 internationally, the level of pain education at medical schools was not consistent with societal needs for pain management.

Teaching methods in pain education must also be evaluated. As reported by both Shipton et al. and Malik et al.,^9,14^ lectures and seminars are the most common pain education methods globally. Other described teaching method included case-based learning, standardized patients, and small group learning. In response to the COVID-19 pandemic, medical education has changed with a high increase of asynchronous learning and virtual class.^7,16^ It has yet to be evaluated whether these new teaching methods will affect not only the number of hours dedicated to pain medicine but also the students' interest for pain medicine with renewed teaching methods, increased schedule flexibility, and greater proportion of self-directed learning.^7^

An additional aspect of pain curricula design is the assessment method at the end of the curriculum. As reported in the review by Shipton et al.,^14^ the most common method of assessment is written examination. In Europe, other reported assessment methods include practical or clinical assessments, presentations, group work, or problem-based learning. Each of these methods is used in less than 10% of European medical schools.^2^ In another review exploring the assessment of medical students in pain medicine, Shipton et al.^15^ highlighted that 80% of included studies assessed students' lower-order cognitive skills (the student “knows” and/or “knows how” ie, remembers, understands, and applies). Only a few studies evaluated higher-order cognitive skills in pain medicine, requiring the student to “show how” by analyzing and evaluating clinical situations through integration of learning skills. In summary, there are numerous proposed programs and curricula for pain education, each reporting a high level of knowledge acquisition^3,6,17^ but very few reporting competencies acquisition.

In conclusion, it remains important to continue assessing efficient teaching methods in pain education to ensure maximum dissemination of pain medicine and pain management knowledge as well as competencies acquisition. The latter may represent the most crucial goal of pain education. With the convenience of the internet, medical knowledge is accessible in various forms. Resources such as Massive Open Online Course, podcasts, and scientific literature may be the reason for the decrease in gain of knowledge between 2009 and 2019 from pain curriculum by Dale et al.^4^ With knowledge so accessible, competency acquisition is the next step. Patients need caregivers that not only “know” but also are able to “show how.”

Publications about pain would do well to assess pain education and pain curricula in comparison with each other. Indeed, what would most enlighten readers would be to compare different pain curricula using Bloom's taxonomy or Miller's clinical competence pyramid^10^ as outcome measures of students' acquisition. This would highlight which education strategies are most efficient for chosen objectives (lower-order or higher-order cognitive skills) and would allow students and educators alike to choose strategies that best fulfill their needs.

## Disclosures

The author has no conflict of interest to declare.

## References

[1] Breivik H Collett B Ventafridda V Cohen R Gallacher D. Survey of chronic pain in Europe: prevalence, impact on daily life, and treatment. Eur J Pain 2006;10:287–333. 16095934 10.1016/j.ejpain.2005.06.009

[2] Briggs EV Battelli D Gordon D Kopf A Ribeiro S Puig MM Kress HG. Current pain education within undergraduate medical studies across Europe: advancing the provision of pain education and learning (APPEAL) study. BMJ Open 2015;5:e006984. 10.1136/bmjopen-2014-006984 PMC4538268 26260345

[3] Cioffi I Dale CM Murphy L Langlois S Musa R Stevens B. Ten years of interfaculty pain curriculum at the University of Toronto: impact on student learning. Pain Rep 2021;6:e974. 34870057 10.1097/PR9.0000000000000974 PMC8635288

[4] Dale CM Cioffi I Murphy L Langlois S Musa R Stevens B. Ten-year mixed method evaluation of pre-licensure health professional student self-reported learning in an interfaculty pain curriculum. Pain Rep 2022;7:e1030. 10.1097/PR9.0000000000001030 PMC9478270 36128043

[5] European Pain Federation. EFIC Pain Curricula. Available at: https://europeanpainfederation.eu/education/pain-curricula/. Accessed June 2022.

[6] Fishman SM Copenhaver D Lorenzen K Schlingmann E Chung C. UC Davis train-the-trainer primary care pain management fellowship: addressing the pain management education gap. Acad Med 2021;96:236–40. 32590468 10.1097/ACM.0000000000003554

[7] Guo MZ Allen J Sakumoto M Pahwa A Santhosh L. Reimagining undergraduate medical education in a post-COVID-19 landscape. J Gen Intern Med 2022;37:2297–301. 35710661 10.1007/s11606-022-07503-7 PMC9202962

[8] IASP. Available at: https://www.iasp-pain.org/Education/Content.aspx?ItemNumber=1698#Centralsensitization. Accessed June 2022.

[9] Malik Z Ahn J Thompson K Palma A. A systematic review of pain management education in graduate medical education. J Grad Med Educ 2022;14:178–90. 35463177 10.4300/JGME-D-21-00672.1 PMC9017274

[10] Miller GE. The assessment of clinical skills/competence/performance. Acad Med 1990;65:S63–7. 2400509 10.1097/00001888-199009000-00045

[11] Pizzo PA Clark NM. Alleviating suffering 101—pain relief in the United States. N Engl J Med 2012;366:197–9. 22256802 10.1056/NEJMp1109084

[12] Pöyhiä R Niemi-Murola L Kalso E. The outcome of pain related undergraduate teaching in Finnish medical faculties. PAIN 2005;115:234–7. 15876496 10.1016/j.pain.2005.02.033

[13] Reid KJ Harker J Bala MM Truyers C Kellen E Bekkering GE Kleijnen J. Epidemiology of chronic non-cancer pain in Europe: narrative review of prevalence, pain treatments and pain impact. Curr Med Res Opin 2011;27:449–62. 21194394 10.1185/03007995.2010.545813

[14] Shipton EE Bate F Garrick R Steketee C Shipton EA Visser EJ. Systematic review of pain medicine content, teaching, and assessment in medical school curricula internationally. Pain Ther 2018;7:139–61. 30058045 10.1007/s40122-018-0103-z PMC6251835

[15] Shipton EE Steketee C Bate F Visser EJ. Exploring assessment of medical students' competencies in pain medicine—a review. Pain Rep 2019;4:e704. 30801044 10.1097/PR9.0000000000000704 PMC6370140

[16] Turbow S Windish DM Lee WW. Unmasking challenges and boosting innovation: the impact of the COVID-19 pandemic on medical education. J Gen Intern Med 2022;37:2144–5. 35562565 10.1007/s11606-022-07666-3 PMC9105579

[17] Watt-Watson J McGillion M Lax L Oskarsson J Hunter J MacLennan C Knickle K Victor JC. Evaluating an innovative eLearning pain education interprofessional resource: a pre-post study. Pain Med 2019;20:37–49. 29931315 10.1093/pm/pny105

